# Permissive Hypercapnia Results in Decreased Functional Vessel Density in the Skin of Extremely Low Birth Weight Infants

**DOI:** 10.3389/fped.2018.00052

**Published:** 2018-03-13

**Authors:** Alexandra Francoise Puchwein-Schwepcke, Kristina Schottmayer, Zuzana Mormanová, Jens Dreyhaupt, Orsolya Genzel-Boroviczeny, Ulrich H. Thome

**Affiliations:** ^1^Divsion of Neonatology, Dr. von Hauner Children’s Hospital, Ludwig-Maximilians-University, Munich, Germany; ^2^Institute for Epidemiology and Medical Biometry, University of Ulm, Ulm, Germany; ^3^Divsion of Neonatology, University Hospital of Leipzig, Leipzig, Germany

**Keywords:** microcirculation, hypercapnia, extremely low birth weight infants, functional vessel density, skin perfusion

## Abstract

**Background:**

Ventilator-induced lung injury with subsequent bronchopulmonary dysplasia remains an important issue in the care of extremely low-birth-weight infants. Permissive hypercapnia has been proposed to reduce lung injury. Hypercapnia changes cerebral perfusion, but its influence on the peripheral microcirculation is unknown.

**Methods:**

Data were collected from 12 infants, who were randomized to a permissive high PCO_2_ target group (HTG) or a control group (CG). Inclusion criteria were birth weight between 400 and 1,000 g, gestational age from 23 to 28 6/7 weeks, intubation during the first 24 h of life, and no malformations. The PCO_2_ target range was increased stepwise in both groups for weaning and was always 15 mmHg higher in the HTG than in the CG. Skin microvascular parameters were assessed non-invasively with sidestream dark field imaging on the inner side of the right arm every 24 h during the first week of life and on the 14th day of life.

**Results:**

Infants in the HTG had significantly higher max. PCO_2_ exposure, which was associated with a significantly and progressively reduced functional vessel density (FVD, *p* < 0.01). Moreover, there were significant differences in the diameter distribution over time, with HTG subjects having fewer small vessels but more large vessels.

**Conclusion:**

High PCO_2_ levels significantly impaired peripheral microcirculation in preterm infants, as shown by a decreased FVD, presumably secondary to peripheral vasoconstriction.

**ISRCTN:**

56143743.

## Introduction

Most extremely low-birth-weight infants (<1,000 g) require respiratory assistance after birth, leading to bronchopulmonary dysplasia (BPD), a chronic form of lung disease (1, 2). Permissive hypercapnia is a strategy of tolerating higher PCO_2_ levels for the benefit of less aggressive ventilation and therefore less mechanical trauma to the lung (3–7). In randomized trials, ventilatory intensities were reduced but resulted in only marginal positive effects on clinical outcome (6, 8–11). Concerns about possible adverse effects such as intraventricular hemorrhage with consecutive developmental impairment, as suggested by retrospective data (12–15), have not been substantiated in randomized controlled trials (6, 8–11). We know that hypercapnia causes dilatation of cerebral arterioles with possibly increased risk of intracranial bleeding (16). On the level of the microcirculation, hypoxia and hypercapnia lead to an increase in perfusion in the cerebral cortex of the rat (17, 18).

In the past 10 years, we have learned that disturbances in the microcirculation have profound effects on outcome. In sepsis, for example, a persistent decrease in functional vessel density (FVD) is indicative of poor survival (19, 20). Changes in the microcirculation were also observed in many different settings such as subarachnoid hemorrhage, congenital heart disease, or myocardial ischemia (21–23). In the preterm infant, a persistent ductus arteriosus (PDA) significantly changes the microcirculation of the skin (24). In preterm hypotensive newborns, FVD in the periphery was significantly elevated in comparison to normotensive infants, suggesting a redistribution of microvascular flow as an underlying mechanism for hypotension (25).

Therefore, the microcirculation is an important, but underrecognized physiologic function with possible impact on important clinical outcome parameters. Furthermore, the microcirculation may be influenced by PCO_2_. We sought to measure the microcirculation in very preterm infants participating in a multicenter randomized controlled trial of different PCO_2_ target ranges (PHELBI) (10). The skin of the preterm infants is a specific and easily accessible organ to observe changes in the microcirculation. Our hypothesis was that the cutaneous microcirculation might be affected by higher PCO_2_ levels during permissive hypercapnia regarding FVD, diameter distribution, and quality of flow.

## Materials and Methods

This study was part of the randomized, prospective multicenter hypercapnia study (PHELBI, ISRCTN 56143743) (10). Infants with gestational ages between 23 and 28 6/7 weeks and birth weights between 400 and 1,000 g, who were intubated during the first 24 h, were randomized within the next 12 h to either a high PCO_2_ target group (HTG) or a control group (CG) after written parental consent. Exclusion criteria were congenital malformations, chromosomal aberrations, and severe asphyxia [pH < 7.0 and heart rate (HR) < 100/min at 10 min of life]. All infants who participated in the PHELBI trial at our center within a predefined time frame of 18 months were included in this study.

During the first 14 days of life, the PCO_2_ target ranges were increased stepwise with the target range in the HTG always 15 mmHg higher than in the CG. PCO_2_ levels were kept between 55 and 65 mmHg in the HTG and 40 and 50 mmHg in the CG during the first 3 days, increased to 60–70 mmHg (HTG) and 45–55 mmHg (CG) on the fourth day, and increased to 65–75 mmHg (HTG) and 50–60 mmHg (CG) again on the seventh day until extubation or peak inspiratory pressure <12 cm H_2_O or day 14. At our center, the skin microcirculation of a subgroup of randomized infants was evaluated as single-center substudy. The institutional review board of the Medical Faculty of Ludwig Maximilians University of Munich approved the study protocol and the consent forms, and all parents granted written informed consent.

Sidestream dark field (SDF) imaging with the handheld video microscope “MicroScan” (Microvision Medical, Wallingford, Pennsylvania) with a five times magnification was used for the assessment of the microcirculation. Monochromatic green light (530 nm) emitted from LEDs, surrounding a light guide, is absorbed by hemoglobin in the erythrocytes and reflected by other tissues or structures. The result is a real-time video of perfused vessels to a depth of 3 mm, thus producing a functional video of the microcirculation. SDF imaging has been validated against conventional capillary microscopy in various studies (26–29). We obtained sequences of 15 s on three different areas on the right inner upper arm. The sublingual region usually preferred for SDF imaging in adults is not feasible in preterm infants. In previous studies, the upper arm has been shown to be easily accessible and to provide a good image quality (24, 25, 30, 31). To reduce artifacts by lanugo hair, we chose the inner side of the arm. Since a PDA influences the skin microcirculation, we consistently used only the right arm to avoid the effects of PDA as a confounder (24).

Measurements started on the first day of life and continued over the first week in 24-h intervals, and the last measurement was obtained on the fourteenth day of life. All measurements were done by one investigator (KS). All video loops were analyzed offline with the microvision analysis software, which offers a semiautomatic analysis. Vessels are automatically detected on the screen, but individual corrections for artifacts such as hair are possible. FVD, which represents total vessel length in millimeter per image area in square millimeter, as well as the distribution of vessel diameters is subsequently calculated. The latter results in the representation of small (<10 μm), medium (10–20 µm), and large (20–100 µm) vessels as a percentage of the total vessel length. Moreover, quality of flow was analyzed, classifying vessels of a quadrant into vessels with “no flow,” “intermittent flow,” “sluggish flow,” “continuous flow,” and “hyperdynamic flow.” Normal flow is defined as continuous flow and hyperdynamic flow as extremely fast flow. No flow and intermittent and sluggish flow stand for different stages of hypodynamic and thus pathological flow. Flow classification was undertaken on the third, fourth, sixth, seventh, and fourteenth day of life and expressed as percentage of vessels with the respective quality of flow. All video analyses were performed with the investigator blinded to patient, treatment, and time point.

Simple statistical analyses were performed with GraphPadPrism 5 (GraphPad Software Inc., La Jolla, CA, USA). For each time point, three video scans per arm were analyzed, and the mean value of a variable was used for statistical analysis. To compare both groups over time, we used linear mixed effects regression models to account for the dependencies of measurements within each infant (fitted with proc mixed, SAS software version 9.4, SAS Institute, Cary, NC, USA). Group comparisons for categorical variables were performed using the chi-square test or Fisher’s exact test as appropriate.

For unpaired comparisons between the two groups, the Mann–Whitney *U* test was used for non-parametric data. For paired comparisons, the Wilcoxon signed-rank test was used.

To consider the development of PCO_2_ exposure between both groups, a further linear mixed effects regression model was used. For other laboratory values (hematocrit, lactate, and CRP), we compared minimal and maximal values over the observation period with the Mann–Whitney *U* test. Because of the exploratory nature of our study, the results of the statistical tests should not be interpreted as confirmatory. An adjustment for multiple testing was not done. *p* < 0.05 was considered as statistically significant.

The PCO_2_ levels were monitored by blood gas analyses, using arterial blood if available, or capillary blood. CRP levels, hemoglobin levels, medication, and blood transfusions were extracted from the charts. Vital signs were recorded before the start of each microcirculation measurement.

## Results

### Clinical Variables

**Figure 1:**
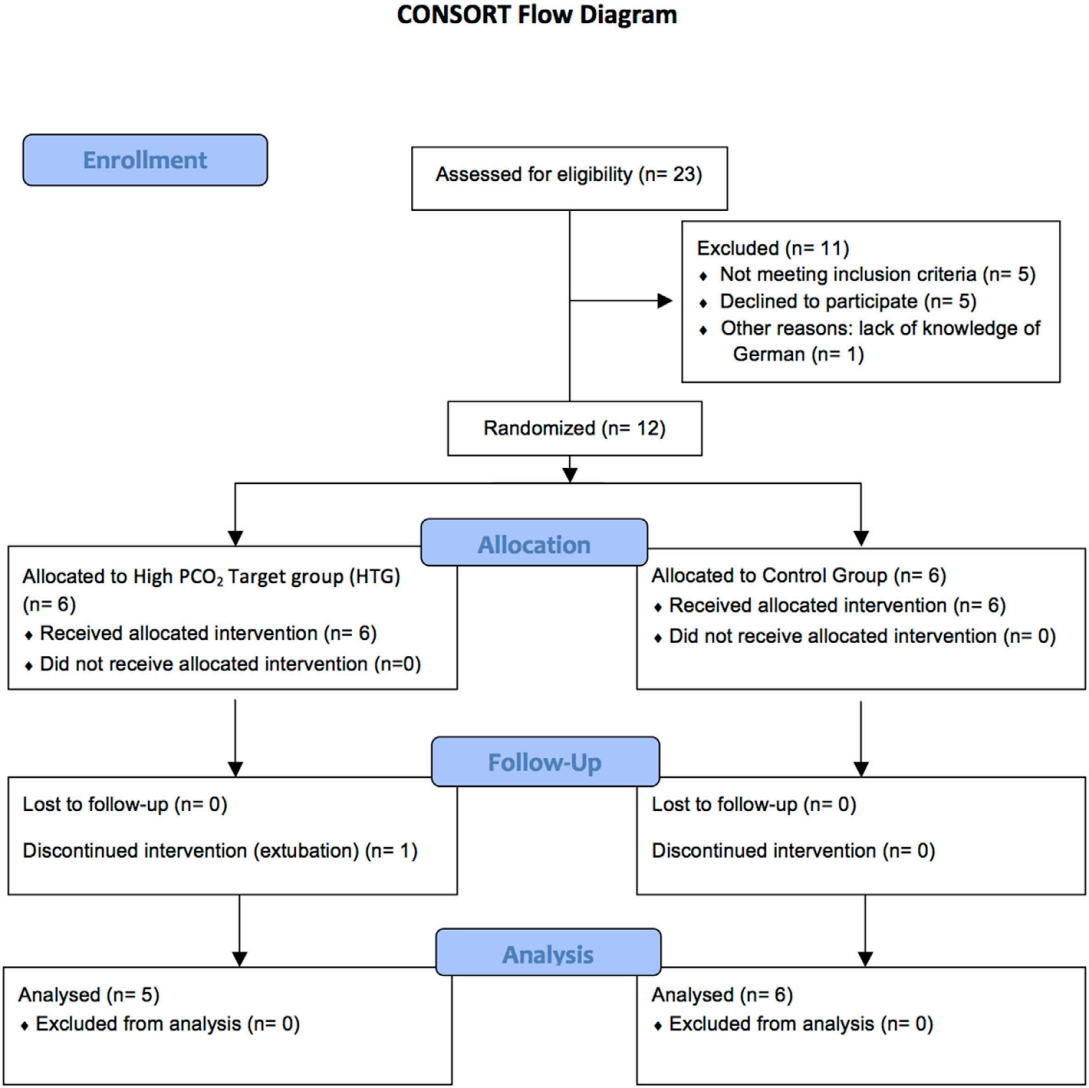
A total number of 12 patients could be included to the study as illustrated in the Consort flow diagram.

**Figure 2:**
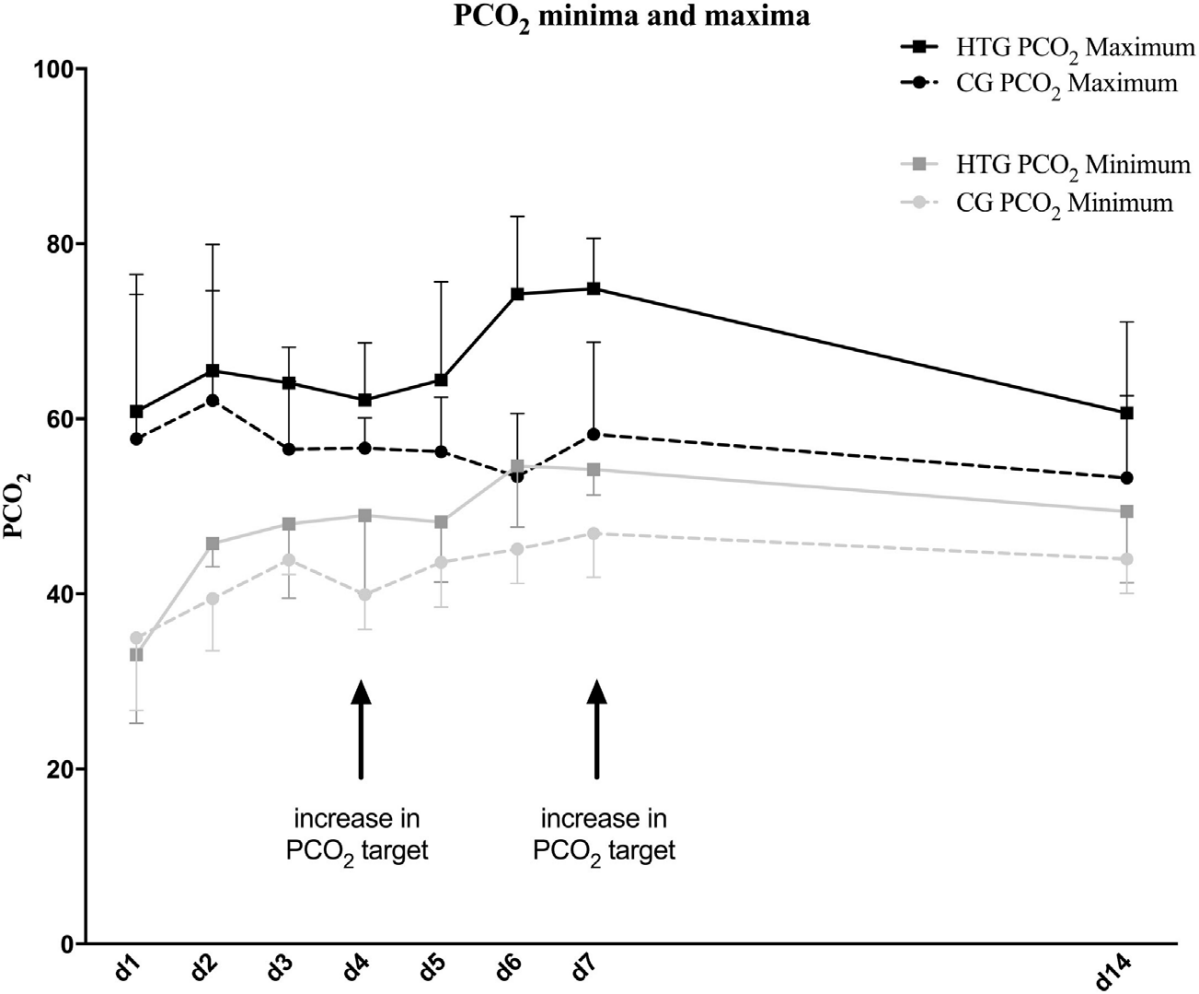
PCO_2_ minima and maxima in the high PCO_2_ target group (HTG) and control group (CG) over the course of 14 days after birth. Minimum and maximum values of all patients are presented as mean values with SDs. PCO_2_ target levels were stepwise increased on the fourth and seventh day of life. As intended, PCO_2_ levels were higher in the HTG.

Twelve preterm infants were enrolled in our study (six from each randomization group, see Figure 1). Gender and birth weight distribution and other variables were similar (Table 1). One infant of the HTG group had to be excluded, as it was extubated on day 2 of life. Another infant of the HTG group died on day 4 of life. Microcirculatory and clinical values of this infant were included as available. Ventilation parameters such as mean airway pressure, positive end-expiratory pressure (PEEP), positive inspiratory pressure (PIP), high-frequency oscillation amplitude, and frequency tended to be lower in the HTG group, as expected, but not significantly. PCO_2_ values were significantly higher (Figure 2; Table 1). Incubator temperature and humidity, HR, and blood pressure did not differ. Other laboratory values, including hemoglobin, hematocrit, lactate, and CRP, did not differ significantly.

**Table 1:** Clinical data.

	High PCO_2_ target group (*n* = 5)	Control group (*n* = 6)	*p* Value
Gestational age (weeks)^a^	24 2/7 (23 3/7–27 6/7)	25 4/7 (24 3/7–27 2/7)	n.s.
Birth weight (g)^a^	685 (590–990)	712 (490–885)	n.s.
Male gender	3 (60%)	3 (50%)	n.s.
Betamethasone	5 (100%)	6 (100%)	n.s.
Tracheal ventilation on day 14	3 (75%)	6 (100%)	n.s.
Average PCO_2_ exposition^b^	56 (53–63)	51 (48–54)	0.03
Catecholamine treatment	1 (20%)	4 (80%)	n.s.
Min. Hct%	31 (29–35)	30 (28–39)	n.s.
Max. lactate (mmol/l)	2.6 (1.3–11.6)	3.2 (1.7–5.8)	n.s.
Max. CRP (mg/l)	0.7 (0.3–3.8)	2.3 (0.2–8.2)	n.s.

*^a^Median (minimum–maximum), Mann–Whitney *U* test*.

*^b^Median (minimum–maximum), Wilcoxon rank sum test*.

*All others: *n* (%), Fisher’s exact test*.

### Microcirculatory Variables

**Figure 3:**
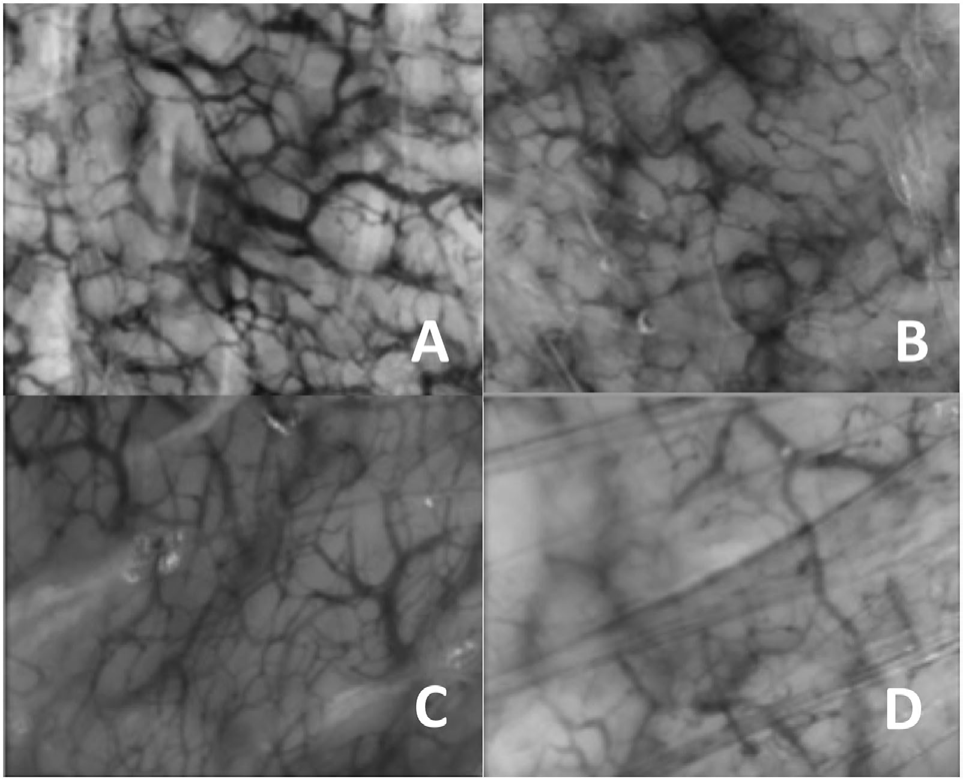
Typical image of the microcirculation. **(A)** Day 1 control group, **(B)** day 14 control group, **(C)** day 1 high PCO_2_ target group, and **(D)** day 14 high PCO_2_ target group.

Images showed similar vasculature at baseline, but declining vessel density toward the end of the study in the HTG (Figure 3). Similar results were determined by the quantitative evaluation.

#### Functional Vessel Density

**Figure 4:**
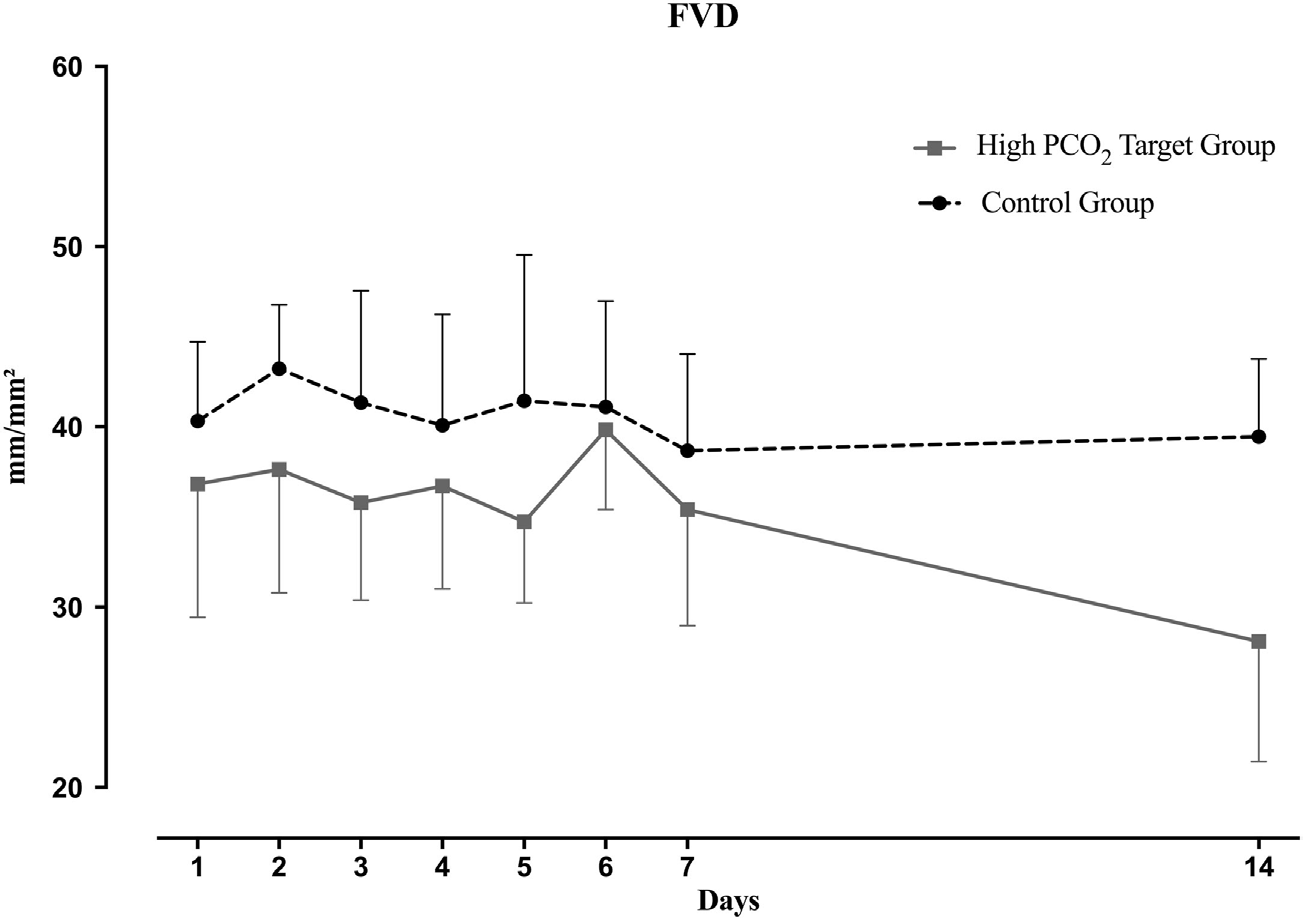
Functional vessel density (FVD) over the course of 14 days. Mean and SD are shown. Overall, FVD was significantly lower in the high PCO_2_ target group (*p* < 0.01 by a linear mixed effects regression model) and time dependent (*p* < 0.01).

**Figure 5:**
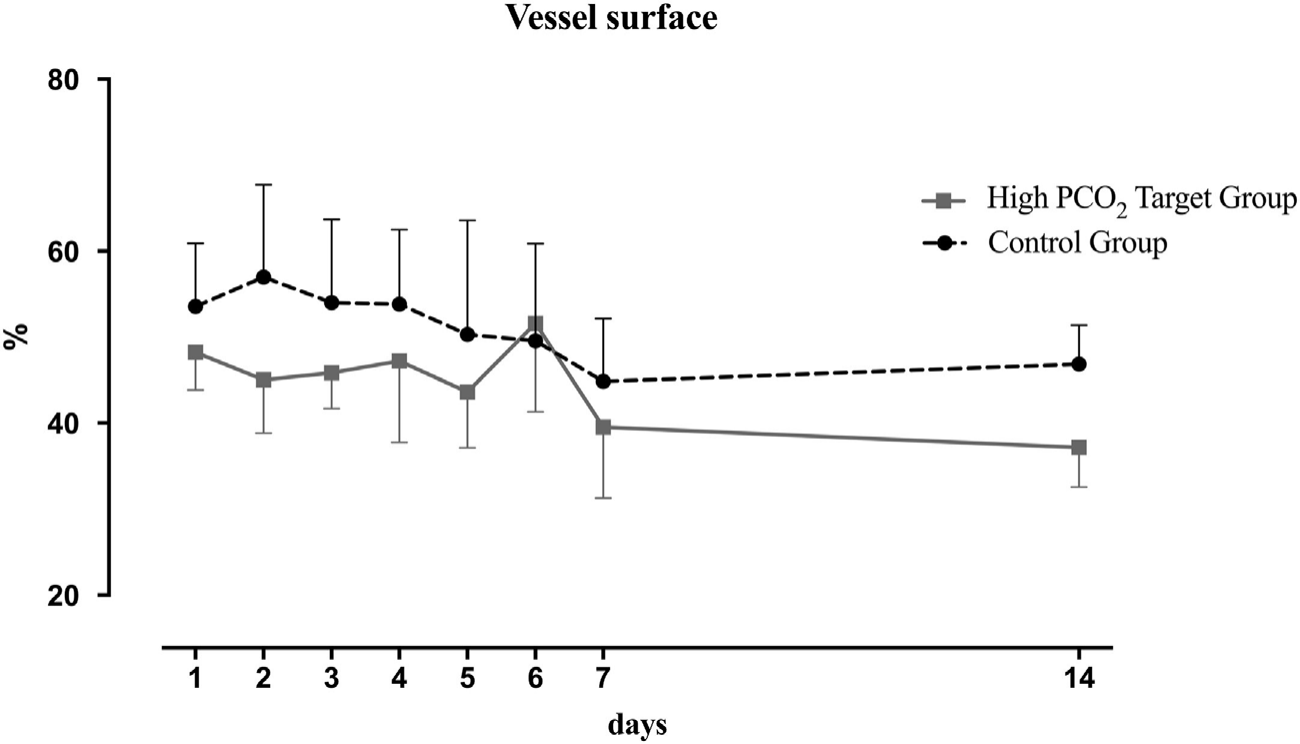
Vessel surface (VS) over the course of 14 days. Mean and SD are shown. Overall, VS was significantly lower in the high PCO_2_ target group (*p* < 0.01 for the between groups comparison, *p* < 0.01 for the time effect).

Functional vessel density did not differ at baseline between HTG and CG, but decreased significantly over the first week in the HTG and remained lower until the end of the study (Figure 4), compared to CG. The comparison between groups and the time effect was significant (both *p* < 0.01). Vessel surface (VS) was also significantly lower in the HTG group over time (Figure 5, *p* < 0.01 for the between-group comparison and *p* < 0.01 for the time effect).

#### Diameter

**Figure 6:**
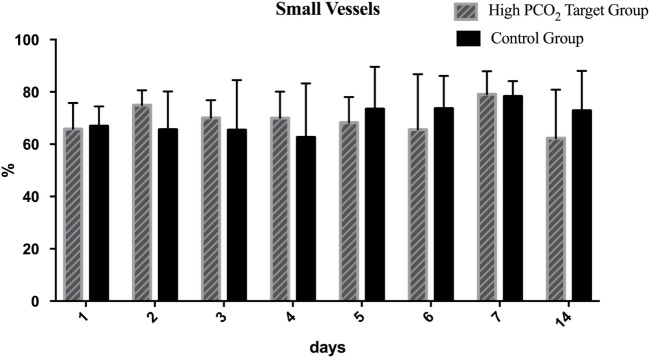
Distribution of small vessels over the course of 14 days. Mean and SD are shown. In the high PCO_2_ target group, the proportion of small vessels significantly decreased resulting in a significant difference between groups in the vessel distribution (*p* < 0.01).

**Figure 7:**
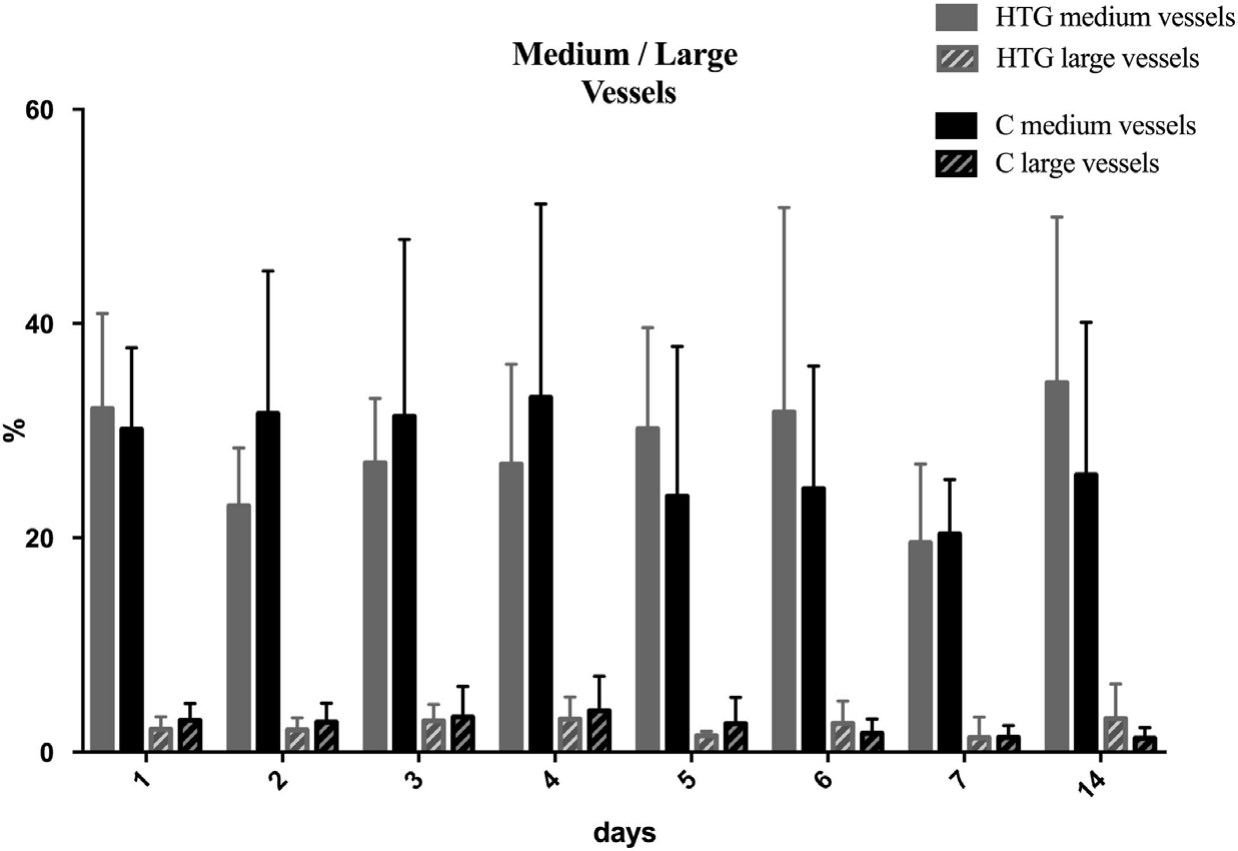
Distribution of medium and large vessels over the course of 14 days. Mean and SD are shown. In the high PCO_2_ target group (HTG), the proportion of medium and large vessels significantly increased over time (*p* < 0.05), resulting in a significant difference between groups in the vessel distribution (*p* < 0.01).

Most detected vessels were small with diameters <10 µm and varied between 60 and 80% of vessel density with no significant difference between the groups at baseline. In the HTG group, the proportion of small vessels significantly decreased (Figure 6), and therefore, the proportion of medium and large vessels increased significantly over time (Figure 7). This resulted in a significant difference between groups in the vessel distribution (*p* < 0.01) with a significant time effect (*p* < 0.05).

#### Flow

The flow in most vessels was continuous, and only a small fraction <6% had no flow in both groups. There were no significant differences between both groups over the time observed. The stepwise increases of PCO_2_ targets on days 4 and 7 were not consistently followed by temporary increases of flow to hyperdynamic levels in all patients (data not shown).

## Discussion

This study demonstrates that 14 days of permissive hypercapnia with relatively high PCO_2_ targets as used in this study have a significant impact on the microcirculation of the skin, compared to the CG with only mildly elevated targets. We demonstrated a significant decrease in FVD, whereas FVD remained constant in the CG. Thus, hypercapnia significantly reduced skin perfusion. The decrease is more prominent in the second week of permissive hypercapnia. This suggests a long-term influence of higher PCO_2_ levels on skin capillaries. This is the first study to evaluate the influence of hypercapnia on the microcirculation of preterm infants over a longer time period (16–18, 32–43). Moreover, we demonstrate significant changes in the diameter distribution with a shift toward larger vessels in the HTG group. At the same time, other clinical parameters as well as ventilation parameters such as PIP and PEEP did not differ between both groups. Thus, the only difference between both groups relevant for our measurements can be seen in the partial pressure of arterial CO_2_.

Peripheral oxygenation is essential for preserving organ function (44). Macrocirculatory parameters are only a poor indicator for tissue oxygen delivery (19). SDF imaging provides a non-invasive technique to visualize peripheral vasoregulation by using a microscope at the bedside.

The reduction of FVD and VS may be the result of a PCO_2_-dependent redistribution of blood flow. Respiratory-induced changes in the partial pressure of arterial CO_2_ also play a major role in the regulation of cerebral blood flow (45). Hypercapnia leads to vasodilatation of cerebral arterioles, whereas hypocapnia leads to vasoconstriction and a subsequent decrease in cerebral arterial flow (46). Therefore, one would have expected a higher FVD in the HTG group, but the opposite effect was found. One possible explanation may be concurrent hypercapnia-induced vasodilatation in deeper areas of the body, including, but not limited, the brain, which may have drawn blood supply away from the skin, leading to a compensatory vasoconstriction and thus explaining the effects observed in this study.

Alternatively, high PCO_2_ may have a direct negative effect on skin perfusion. Both suggested mechanisms would lead to a redistribution of the blood volume toward larger vessels in the deeper areas.

Another possible explanation for a reduction in FVD and VS may be the so-called Bohr effect. Raised PCO_2_ levels and a low pH reduce the oxygen affinity of hemoglobin and facilitate oxygen unloading, thus enhancing oxygen diffusion from hemoglobin to the tissues (32, 34). Therefore, the lower FVD does not need to be a negative effect; it could also be a sign of better tissue oxygenation while reducing the need for perfused blood vessels.

A fourth possible explanation for the reduction in FVD may be vasodilation due to high PCO_2_ values, which could explain the larger proportion of medium and large vessels observed in our HTG group. As a compensatory mechanism, small vessels would constrict and would result in a reduced overall FVD and VS due to hypercapnia.

Therefore, the significant changes in diameter distribution seem to be another interesting finding in our study. After birth, both groups showed a similar distribution of vessel diameter. When analyzed on the fourteenth day, the fraction of small vessels had decreased in the HTG, while it increased in the CG. In return, medium and large vessels were more prominent in the HT group.

Komori et al. (36) described proportionate increasing diameters and velocity while increasing PCO_2_ up to levels of 80 mmHg in rabbits. Hudetz et al. (18) obtained similar results when studying the effects of hypercapnia in the capillaries of the rat cerebral cortex. The increasing diameters are consistent with our observation that the proportion of medium and large vessels increases with higher PCO_2_ levels. However, we cannot confirm the increased velocity, because even though the fraction of hyperdynamic flow appeared to increase with higher PCO_2_ targets, this did not reach statistical significance in our study. In sepsis models, similar microcirculatory changes indicated a poorer outcome (47, 48).

Due to the strict exclusion criteria and the very time-consuming methods and analyses, our study population was rather small and random errors cannot be ruled out. Another limitation might be that analyses of the flow were performed semiquantitatively.

The microcirculation is influenced by various factors, such as hematocrit and hemoglobin level, incubator temperature, blood transfusions, infections, PDA, and gender (16, 18, 24, 28, 31, 49–52). There were no significant differences in our groups concerning these possible confounders. Blood pressure and HR showed no significant or only individual influence on the microcirculation in previous studies (30, 53) and were similar in both groups. Catecholamine treatment has been shown to increase FVD in premature infants (25), which might be a possible confounder in our results. Catecholamine treatment appeared to occur more frequently in the CG than the HTG, although this has not been statistically significant. However, catecholamines were given only for a few days. Effects of catecholamine treatment on the microcirculation have been demonstrated only in the first 6 h after birth and not after discontinuation of the treatment. Therefore, we do not think that catecholamine treatment confounded our results, showing long-term reduction in FVD seen in the HTG.

Another possible limitation is the lack of measurements between the seventh and the fourteenth day. We had decided beforehand to concentrate on the immediate effects after elevation of PCO_2_ as well as the long-term effects under treatment with permissive hypercapnia and not to focus on single data points.

Vascular development is a key process in lung development, and impaired vascular development has been linked to the emergence of BPD. Due to our small patient group, we were unable to detect significant differences in clinical outcome. However, if we look at the missing benefit or even worsened pulmonary outcome of the patients of the HTG group in the PHELBI trial (10), we speculate that the pulmonary outcome may have been linked to the differences in microcirculation. If lung vessels respond in the same manner as skin vessels, the reduced vessel density after prolonged hypercapnia observed in this study may be one possible additional mechanism why permissive hypercapnia as performed in this study has not fulfilled the hope of reducing the incidence of BPD (10). Although flow was not significantly impaired in our study, which might be due to the small number of patients, a reduction in FVD results in a pronounced transformation of microvascular architecture, which could be an important pathophysiological mechanism in the genesis of BPD.

In conclusion, our study showed a significant decrease in FVD after long-term treatment with high PCO_2_ targets, possibly due to flow redistribution toward larger vessels in deeper areas. The microcirculation is a sensitive parameter that reacts to changes in multiple diseases or conditions. In our case, it provided a different perspective on the benefits and disadvantages reported under permissive hypercapnia (9) and should be further explored in larger studies.

## Ethics Statement

This study was carried out in accordance with the recommendations of the institutional review board of the Ludwig Maximilians University with written informed consent from all subjects. All subjects gave written informed consent in accordance with the Declaration of Helsinki. The protocol was approved by the institutional review board of the Ludwig Maximilians University.

## Author Contributions

AP-S, KS, ZM, OG, and UT conceived and planned the trial and the measurements. KS and ZM carried out the measurements. KS did the analysis of the microcirculatory videos and clinical data. AP-S, KS, ZM, OG, JD, and UT contributed to the interpretation of the results. JD, UT, and AP-S performed the statistical analyses. AP-S took the lead in writing the manuscript. All authors provided critical feedback and helped shape the investigations, analyses, and the manuscript.

## Conflict of Interest Statement

The authors declare that the research was conducted in the absence of any commercial or financial relationships that could be construed as a potential conflict of interest.

## Abbreviations

A/C, assisted/controlled; AUC, area under the curve; BPD, bronchopulmonary dysplasia; ELBI, extremely low birth weight infant; SDF, sidestream dark field; FVD, functional vessel density; HTG, high PCO_2_ target group; HFO, high-frequency oscillation; HR, heart rate; IVH, intraventricular hemorrhage; MAP, mean airway pressure; CG, control group; OPS, orthogonal polarization spectral; PDA, persistent ductus arteriosus; PEEP, positive end-expiratory pressure; PIP, positive inspiratory pressure; SIMV, synchronized intermittent mandatory ventilation; VS, vessel surface.
